# Conditioned media of pancreatic cancer cells and pancreatic stellate cells induce myeloid-derived suppressor cells differentiation and lymphocytes suppression

**DOI:** 10.1038/s41598-022-16671-9

**Published:** 2022-07-19

**Authors:** Yuen Ping Chong, Evelyn Priya Peter, Feon Jia Ming Lee, Chu Mun Chan, Shereen Chai, Lorni Poh Chou Ling, Eng Lai Tan, Sook Han Ng, Atsushi Masamune, Siti Aisyah Abd Ghafar, Norsharina Ismail, Ket Li Ho

**Affiliations:** 1 School of Postgraduate Studies, International Medical University, Kuala Lumpur, Malaysia; 2 School of Pharmacy, International Medical University, No. 126, Jalan Jalil Perkasa 19, Bukit Jalil, 57000 Kuala Lumpur, Malaysia; 3 Division of Gastroenterology, Tohoku University Graduate School of Medicine, Sendai, Japan; 4 Department of Basic Science and Oral Biology, Faculty of Dentistry, Universiti Sains Islam Malaysia, Seremban, Malaysia; 5 Natural Medicines and Products Research Laboratory, Institute of Bioscience, Universiti Putra Malaysia, Serdang, Selangor Malaysia

**Keywords:** Cancer, Cell biology, Immunology

## Abstract

As pancreatic cancer cells (PCCs) and pancreatic stellate cells (PSCs) are the two major cell types that comprise the immunosuppressive tumor microenvironment of pancreatic cancer, we aimed to investigate the role of conditioned medium derived from PCCs and PSCs co-culture on the viability of lymphocytes. The conditioned medium (CM) collected from PCCs and/or PSCs was used to treat peripheral blood mononuclear cells (PBMCs) to determine CM ability in reducing lymphocytes population. A proteomic analysis has been done on the CM to investigate the differentially expressed protein (DEP) expressed by two PCC lines established from different stages of tumor. Subsequently, we investigated if the reduction of lymphocytes was directly caused by CM or indirectly via CM-induced MDSCs. This was achieved by isolating lymphocyte subtypes and treating them with CM and CM-induced MDSCs. Both PCCs and PSCs were important in suppressing lymphocytes, and the PCCs derived from a metastatic tumor appeared to have a stronger suppressive effect than the PCCs derived from a primary tumor. According to the proteomic profiles of CM, 416 secreted proteins were detected, and 13 DEPs were identified between PANC10.05 and SW1990. However, CM was found unable to reduce lymphocytes viability through a direct pathway. In contrast, CM that contains proteins secreted by PCC and/or PSC appear immunogenic as they increase the viability of lymphocytes subtypes. Lymphocyte subtype treated with CM-induced MDSCs showed reduced viability in T helper 1 (Th1), T helper 2 (Th2), and T regulatory (Treg) cells, but not in CD8^+^ T cells, and B cells. As a conclusion, the interplay between PCCs and PSCs is important as their co-culture displays a different trend in lymphocytes suppression, hence, their co-culture should be included in future studies to better mimic the tumor microenvironment.

## Introduction

Pancreatic ductal adenocarcinoma (PDAC) is one of the top leading causes of cancer-related deaths worldwide and it is projected to be one of the top three cancer killers in year 2030^1–3^. Early diagnosis of pancreatic cancer could lead to a better prognosis. However, patients with pancreatic cancer are usually asymptomatic or display very mild, non-specific symptoms^2,4–6^. Consequently, patients are usually diagnosed at a later stage when local invasion or metastasis of disease is present, which is not curable with surgery and has a high recurrence rate^7,8^.

A unique characteristic of PDAC tumor is its dense stromal layer, also known as a desmoplastic reaction^9,10^. This layer is mainly composed of extracellular matrix (ECM) components, which usually account for more than half of the total tumor volume. ECM is a major product of pancreatic stellate cell (PSC), which can be found in both healthy and cancer patients^2,9,11,12^. However, in response to pathological stimuli, quiescent PSC will be activated and produce excessive ECM proteins, leading to the creation of a pro-tumoral microenvironment^9,13–18^. Besides, activated PSC also secrete matrix degrading enzymes such as matrix metalloproteinases, which contribute to basement membrane destruction that enhances local invasion^8,15,18–21^. As reported, pancreatic cancer cell (PCC) is a major contributor to PSC activation, in which the secreted proteins of PCC can enhance the activation and proliferation of PSC, thereby facilitate local or distant tumor invasion^22^.

Other than the dense stroma layer, PDAC tumor is unique because of the tumor microenvironment (TME) that is rich in immune cells. Despite the abundant immune cells in the TME, PDAC tumor is still highly immunosuppressive due to the dysfunctional immune cells. Immune suppression can be induced thru multiple mechanisms, which include the recruitment of suppressor cells, secretion of suppressive cytokines, and expression of cell-surface proteins that confer inhibitory signals to the effector immune cells^23–25^. In addition to the direct inhibitory effects on antitumor immune response, tumor-derived cytokines promote the differentiation of suppressor cells, which secrete cytokines that disrupt the immune balance by inducing pro-tumoral immune response^26^.

Myeloid-derived suppressor cell (MDSC) is the major population of suppressor cells in PDAC^27^. They are a group of immature myeloid cells derived from bone marrow that exist in a healthy individual at a level as low as 0.5% of the peripheral blood mononuclear cells (PBMCs), which could increase by tenfold in cancer patients^27–29^. Interestingly, tumor-derived factors such as granulocyte–macrophage colony-stimulating factor (GM-CSF), interleukin (IL) 3, and vascular endothelial growth factor (VEGF) are responsible for MDSC accumulation and expansion; while stromal- or activated T cells-derived factors such as IL-1β, IL-6, and prostaglandins are mostly responsible for MDSC activation^26,28^. As reported by Mace et al., the medium conditioned by PSCs could induce the differentiation of immature MDSCs in PBMCs into matured MDSCs that portrayed T cell suppressive property^30^. However, the specific growth factors that were involved have remained unclear.

As shown in the previous studies, the individual effect of PCC and PSC in suppressing lymphocytes have been studied extensively, which is either directly by secreting the suppressive cytokines, or indirectly by promoting the differentiation of suppressive MDSC^31–36^. However, the combinatory effect of PCC- and PSC-secreted proteins on lymphocytes is yet to be investigated. As PCC and PSC are the two most abundant cell types that build the TME of PDAC, we should study their effects on lymphocytes in a co-culture to better mimic the PDAC TME. In this study, we hypothesized that PCC- and PSC- secreted proteins can induce lymphocyte cell death, either through a direct (suppressive secreted proteins) or through an indirect (induce MDSC differentiation) pathway. The hypothesis was tested by analyzing the viability of lymphocytes after treated with conditioned media (CM) or CM-induced MDSC. Besides, we also hypothesized that the PCCs derived from primary and metastatic tumors may interact differently with PSC and thus lead to different levels/patterns of lymphocytes suppression. This study would elucidate the immunosuppressive nature of PDAC TME and make future development of immunotherapy possible^27,30,35^.

## Materials and methods

### Cell line and cell culture

The human PCC cell lines PANC 10.05 and SW1990 were obtained from American Type Culture Collection (Manasas, VA, USA). Cells were maintained in Dulbecco’s Modified Eagle’s medium (DMEM) supplemented with 10% heat-inactivated FBS, and 1% penicillin and streptomycin (Nacalai Tesque, Japan). The immortalized human PSC line, hPSC21-S/T was derived from a resected pancreas from a patient that was undergoing surgery for pancreatic cancer^11^. They were maintained in Dulbecco’s Modified Eagle medium/Ham’s F-12 (Ham’s F12) supplemented with 10% heat-inactivated FBS, and 1% penicillin and streptomycin. During the experiment, PCCs and PSCs were cultured in 1:1 ratio of DMEM: DMEM/Ham’s F12.

The study has been divided into two phases. In phase I, peripheral blood mononuclear cells were treated with media conditioned by PCCs and PSCs at different ratios. Culture 1 (C1) comprises PCCs (PANC 10.05 or SW1990) only, Culture 2 (C2) comprises PCCs: PSCs at different ratios, and Culture 3 (C3) comprises PSCs only. Different ratios of PCCs: PSCs at 20:80, 40:60, 60:40, 80:20 were used in C2.

In phase 2, lymphocyte subtypes were treated with conditioned media collected from C1, C3, and Culture 4 (C4) that comprises PCCs: PSCs at a ratio of 1:1.

### Ethical approval

Written informed consent was obtained from all volunteers that have donated blood for PBMC isolation and the following experiments. The protocol of this study was approved by Joint Committee on Research Ethics, International Medical University, Malaysia.

### Peripheral blood mononuclear cell (PBMC) isolation

Whole blood was donated by volunteers and collected in Vacutainer^®^ blood collection tubes with anticoagulant (EDTA or heparin). The blood was then layered on top of histopaque-1077 (Sigma-Aldrich, USA) in a 1:1 ratio and centrifuged for 30 min at 400×*g*. After centrifugation, the opaque interface containing the mononuclear cells was aspirated and washed with phosphate buffered saline solution (PBS) thrice. After the last wash, supernatant was discarded, and the pellet was resuspended with 1 mL of culture medium.

### Conditioned medium (CM) collection

Group C1–C4 were seeded at a total density of 1.5 × 10^5^ cells/well in 6-well culture plates (Eppendorf, Germany). Cells were incubated for 3 days, and the CM was collected and stored at -80 °C.

### Phase I: treatment of PBMCs with CM

In phase I, PBMCs were treated with CM collected from group C1–C3, and their total lymphocytes population was assessed using flow cytometry analysis. PBMCs (2 × 10^6^ cells/well) were seeded in 6-well culture plates and CM was added to achieve a concentration of 10% (total volume per well = 3 mL). As each cell line had a different growth rate, normalization was performed (formula shown below) to adjust the final volume of CM used to treat the PBMCs. This would avoid potential bias due to the difference in concentration of secreted proteins in CM (CM from groups with a lower cell number will have lower concentration of secreted proteins from PCCs and/or PSCs).

$$\begin{aligned} Normalized \;CM \;volume &= \frac{standard \;cell\; number* \times 300\, \upmu L}{{Number \;of \;cells}}\\ *standard\; cell \;number &= cell\; number \;for \;the\; group \;with \;the \;highest\; number\; of\; cells \end{aligned}$$

Cells were cultured for 7 days with medium changed on day 3. After 7 days, the cells were collected and processed for flow cytometry analysis.

### Flow cytometry

After 7 days of treatment with CM, PBMCs were analyzed by flow cytometry and the total lymphocytes population was identified based on cell size and granularity. The viability dye, 7-aminoactinomycin D (7AAD) was used to exclude dead cells that may lead to false-positive results.

### Proteomic analysis

#### Conditioned media collection and concentration

Both PCC lines were seeded at a total density of 1.5 × 10^5^ cells/well in 6-well culture plates (Eppendorf, Germany) and incubated for 48 h. Next, the media were replaced with serum free media. After 24 h, the CM were collected and concentrated using the Pierce™ Protein Concentrator with PES membrane and molecular weight cut-off at 3 kDa (ThermoFisher Scientific, USA).

#### Protein digestion and LC–MS/MS

The concentrated CM were subjected to protein digestion with MS grade trypsin (Merck, Germany) using dithiothreitol (DTT) (Sigma-Aldrich, USA) as reducing agent and iodoacetamide (IAM) (Merck, Germany) as alkylating agent. After protein digestion, the samples were desalted with Pierce™ C18 Tips (ThermoFisher Scientific, USA) prior to drying using vacuum concentrator (Eppendorf, Germany). The lyophilized samples were reconstituted in 0.1% formic acid in H_2_O and run in an Agilent 1200 HPLC coupled with Agilent 6550 iFunnel Q-TOF LC/MS (Agilent Technologies, USA).

#### Differentially expressed proteins (DEPs) identification and GO terms analysis

Raw data was processed with Peaks X+ and DEPs were identified using RStudio v.2022.02.2 + 485 and DEBrowser v.1.22.5. Proteins with false discovery rate (FDR) < 0.05 and fold change (FC) ≥ 2 were classified as significantly differentially expressed. Gene ontology (GO) enrichment analysis was performed on the DEPs using DEBrowser, GO terms with FDR < 0.05 were considered significantly enriched.

### Phase II: treatment of CD4^+^, CD8^+^ T cells, and B cells with CM and CM-induced MDSC

#### CD4^+^ T cells isolation and differentiation

Naïve CD4^+^ T cells were isolated from PBMCs using an immunomagnetic negative selection kit (Stemcell Technologies, Canada). The isolation was performed according to the manufacturer’s protocol. At the end of isolation, the naïve CD4^+^ T cells fraction was divided into 3 portions. Each portion was cultured in ImmunoCult™-XF T Cell Expansion Medium supplemented with ImmunoCult™ Human CD3/CD28/CD2 T Cell Activator and the differentiation supplement cocktail for T helper 1 cells (Th1), T helper 2 cells (Th2), and T regulatory cells (Treg) respectively (Stemcell Technologies, Canada). Th1 and Treg was incubated for 7 days for activation, whereas Th2 was incubated for 14 days. Medium was changed every 2–3 days with the density maintained at 1 × 10^6^ cells/mL. All cultures were maintained in 37 °C incubator, 5% CO_2_.

#### CD8^+^ T cells isolation

CD8^+^ T cells were isolated directly from whole blood using an immunomagnetic negative selection kit (Stemcell Technologies, Canada) according to manufacturer’s protocol. The isolated CD8^+^ cells were cultured in ImmunoCult™-XF T Cell Expansion Medium supplemented with ImmunoCult™ Human CD3/CD28/CD2 T Cell Activator for 9 days, with medium changing every 2–3 days and density adjusted according to manufacturer’s recommendations (Stemcell Technologies, Canada).

#### B cells isolation

B cells were isolated from whole blood directly using an immunomagnetic negative selection kit (Stemcell Technologies, Canada) according to the manufacturer’s protocol. The isolated B cells were then seeded into a 96-well plate in complete DMEM: DMEM/Ham’s F12 medium.

#### MDSCs isolation

CM collected from culture groups C1, C3, and C4 were used to treat isolated PBMCs for 7 days to induce MDSCs differentiation. As a control, PBMCs were also seeded without CM treatment to access the suppressive properties of uninduced MDSCs. On day 7, the uninduced and CM-induced MDSCs were isolated using an immunomagnetic positive selection isolation kit (Stemcell Technologies, Canada). Isolated MDSCs were then seeded in 96-well plate at a density of 0.25 × 10^4^ cells per well and incubated overnight.

#### Treatment of isolated lymphocytes with CM and MDSCs

In phase II, lymphocyte subtypes were treated with CM (direct pathway) and CM-induced MDSCs (indirect pathway) using the CM collected from group C1, C3, and C4, and the cell viability was assessed using a microplate reader.

For the lymphocytes that were treated with CM, activated CD4^+^, CD8^+^ T cells, and B cells were seeded into 96-well plate and treated with medium conditioned by group C1, C3, and C4 at the concentrations of 10%, 20% and 30%. Untreated lymphocyte subtypes were seeded as control. The cell viability was then assessed using CellTiter-Glo^®^ Luminescent Cell Viability Assay at 48 h (Promega, USA).

As for the lymphocytes that were treated with MDSCs, activated Th1, Th2, Treg, CD8^+^ T cells, and B cells were seeded into the wells with MDSCs at a density of 0.25 × 10^4^ cells per well. Untreated lymphocyte subtypes and untreated MDSCs (both uninduced and induced by CM) were also seeded as controls. All groups were incubated for 48 h, and at the end of incubation, CellTiter-Glo^®^ Luminescent Cell Viability Assay was used to measure the viability (Promega, USA). The viability of lymphocyte subtypes after treatment was calculated according to the following formula.

$$Viability \;of\; lymphocyte = \frac{Total\;viability - viability\; of\; untreated\; MDSC}{{Viability\; of \;untreated \;lymphocyte \;subtypes }}$$

### Statistical analysis

All experiments were performed in triplicates and statistical analysis was performed using Statistical Package of Social Sciences (SPSS) software (version 25). Analysis of Variance (ANOVA) was carried out, followed by Duncan post-hoc test to analyze the differences among groups. A p-value less than or equal to 0.05 was considered significant.

### Ethics approval

The research has been approved by Ethical Board of International Medical University and the whole research process complies with the principles of the Declaration of Helsinki.

### Consent to participate

Written informed consent was obtained from the participants of the study.

## Results

### Effects of PCCs and PSCs CM on lymphocytes populations

**Figure 1:**
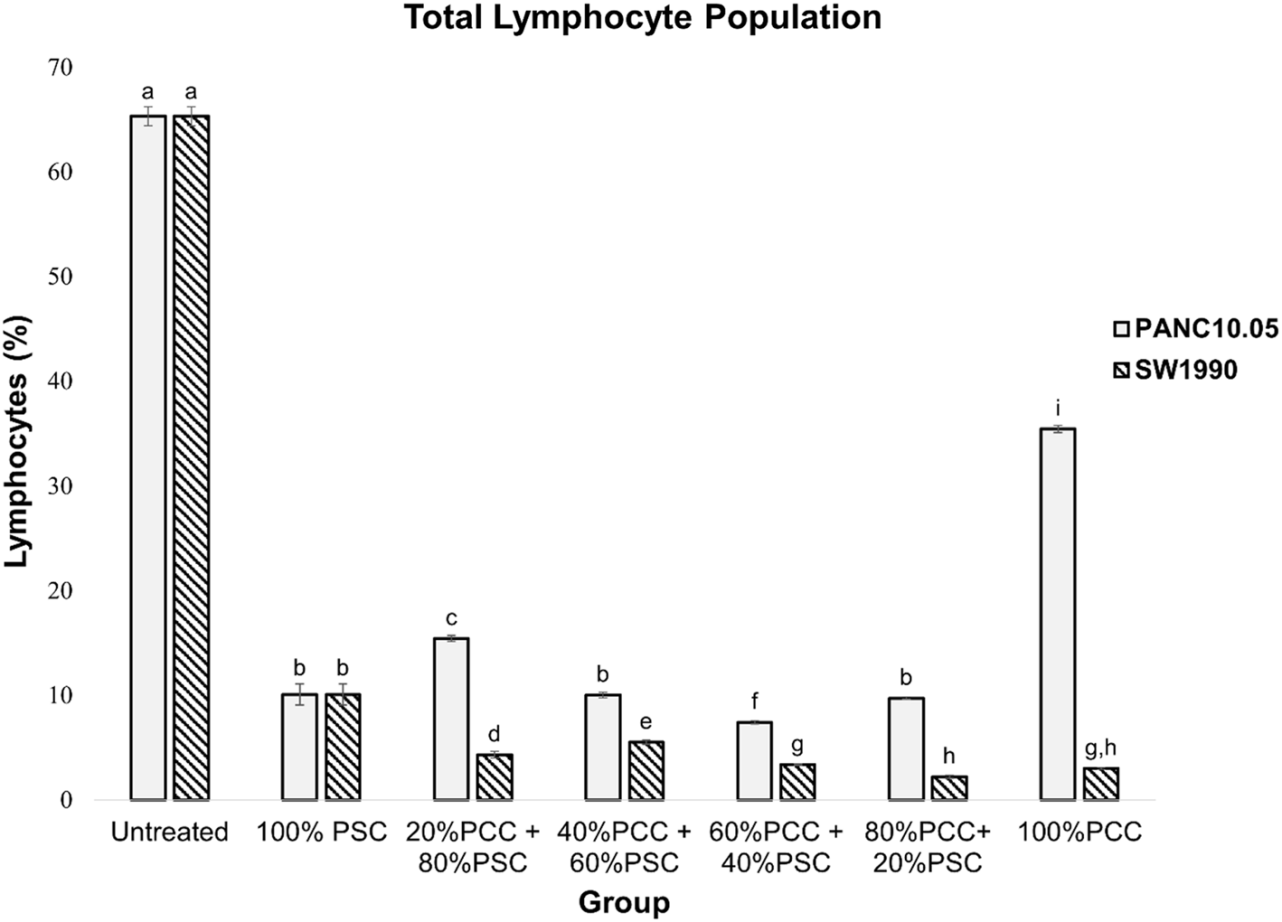
The percentage of lymphocytes in CM treated PBMCs. PBMCs were isolated and treated with medium conditioned by PCCs and/or PSCs for 7 days. Percentage of lymphocytes was determined to verify the lymphocytes suppression of CM on lymphocytes. Statistical significance is indicated by the letters above each column, in which the columns that do not share a common letter have a significance of p ≤ 0.05.

Figure 1 shows the percentage of lymphocytes after 7 days of CM treatments. All CM treated groups had a lymphocytes percentage that was at least 50% lower than the untreated group. Noteworthy, 100% PANC10.05 CM treated group had a lymphocytes percentage that was at least 2 times higher than other treated groups. These results suggested that the secreted proteins in the CM were able to induce lymphocytes suppression, in which a stronger suppression was observed in the SW1990 treated group as compared to the PANC10.05 treated group.

### The DEPs in PCC lines

In order to determine the potential proteins responsible for the different suppressive properties of PANC10.05 and SW1990, CM that contains the secreted proteins were analyzed using LC–MS/MS.

**Figure 2:**
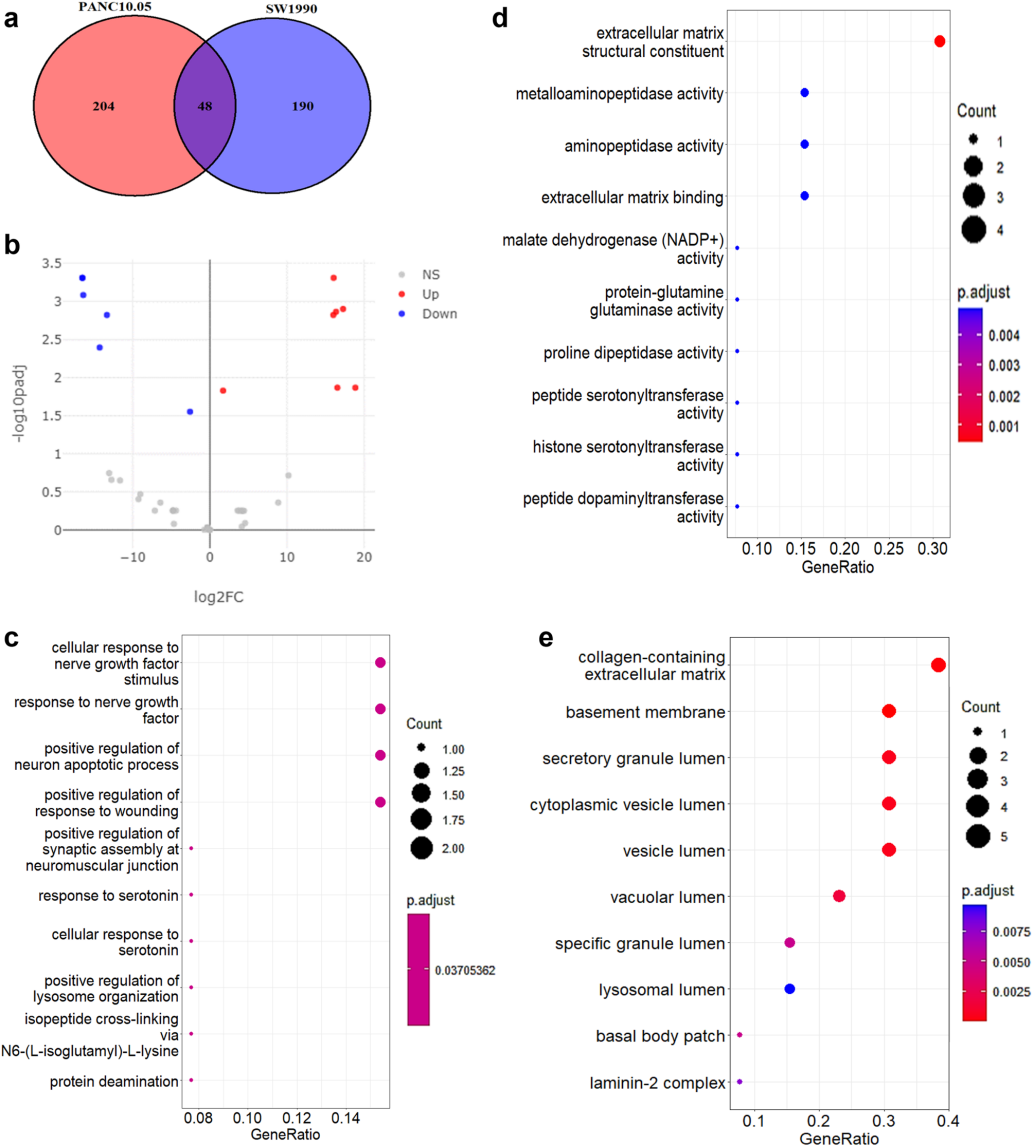
The proteomic profiling of PANC10.05 and SW1990. (**a**) Venn diagram for the number of proteins expressed by each cell line. Number in the intersection represents the number of expressed proteins shared by PANC10.05 and SW1990. (**b**) Volcano plot of the DEPs between PANC10.05 and SW1990. (**c**–**e**) Gene Ontology enrichment analysis of differentially expressed proteins (DEPs). X-axis represents the gene ratio and Y-axis represents the corresponding GO term. (**c**) Ontology = Biological Process; (**d**) Ontology = Molecular Function; (**e**) Ontology = Cellular Component.

Figure 2a shows a Venn diagram that represents the number of secreted proteins detected in each PCC line CM. Furthermore, a Volcano plot that serves as a visual tool for the overall protein expression was generated using the log2 FC score and − log10 padj (Fig. 2b). In total, 13 DEPs were found based on the cut-off criteria (padj < 0.05 and FC ≥ 2), in which 6 proteins were upregulated in PANC10.05 and 7 proteins were upregulated in SW1990 (Table 1). Furthermore, GO enrichment analysis was performed as shown in Fig. 2c–e. The DEPs were significantly enriched in biological processes containing cellular response to nerve growth factor stimulus, response to nerve growth factor, and positive regulation of neuron apoptotic process (Fig. 2c). In terms of molecular function, the DEPs were related to extracellular matrix structural constituent, metalloaminopeptidase activity, and aminopeptidase activity (Fig. 2d). Whereas for cellular components, the enriched GO terms were associated with collagen-containing extracellular matrix, basement membrane, and secretory granule lumen (Fig. 2e).

**Table 1:** The DEPs detected between PANC10.05 and SW1990.

Upregulated by	Protein	Fold change	padj	Functions
PANC10.05	NPEPPS	− 16.52	0.0005	Enhances cisplatin resistance^70^
	EEF2	− 16.52	0.0005	Plays an oncogenic role in cancer growth^71^ MHC-class I-binding eEF2 peptide induces antitumor cytotoxic T lymphocyte responses^72^
	MDH2	− 16.40	0.0008	MDH2 depletion causes toxicity to tumor cells, however the effect is moderate^73^
	PEPD	− 13.34	0.0015	Regulator of p53 tumor suppressor^74^ Elimination of PEPD causes tumor cell death via the activation of p53^74^
	GRN	− 14.28	0.0040	Correlates negatively with MHC class I expression and CD8^+^ T cell infiltration^75^ Inhibition of GRN restores the MHC class I expression on PDAC cells^75^
	ACTG1	− 2.57	0.0280	Associates with poor prognosis^76^ Regulates the cell proliferation and migration via ROCK signaling pathway^77^
SW1990	LAMC2	16.05	0.0005	Associated with poor prognosis, tumor stage and subtypes^78^ Regulates gemcitabine sensitivity through EMT and ABC transporters in PDAC^79^
	HSPG2	17.27	0.0013	Regulate cell adhesion, cell migration and endocytosis^80^ Modulate cancer-associated fibroblasts and the effects of cytokines on infiltrating cells such as macrophages^81^
	AGRN	16.35	0.0014	Facilitate the cancer cell growth, invasion, and migration^82^
	TGM2	16.00	0.0015	Involved in PI3K/Akt survival pathway, TGF-β signaling pathway, and NF-κB signaling pathway activation^39^ A potential marker for the immunosuppressive Th2-IL4-activated macrophages (M2)^39^
	TGFβI	18.86	0.0135	Secreted predominantly by tumor-associated macrophages^83^ Inhibit CD8^+^ T cell responses via the inhibition of TCR signaling^84^
	PTX3	16.53	0.0135	Regulates complement activation and tumor promoting inflammation^85^ Inhibition of PTX3 increases macrophage infiltration, pro-inflammatory cytokine production, and complement activation^86^
	LCN2	1.71	0.0148	An innate immune protein that promotes inflammation^87^ Protect MMP9 from degradation^87^ Stimulate a pro-inflammatory response in PSCs^88^

### Effects of CM on isolated T lymphocytes

As mentioned in section “Effects of PCCs and PSCs CM on lymphocytes populations”, lymphocytes suppression was observed when PBMCs were treated with CM. Hence, we isolated the subtypes of lymphocyte and treated them with CM to further investigate if the CM is inducing lymphocytes suppression via the direct pathway.

**Figure 3:**
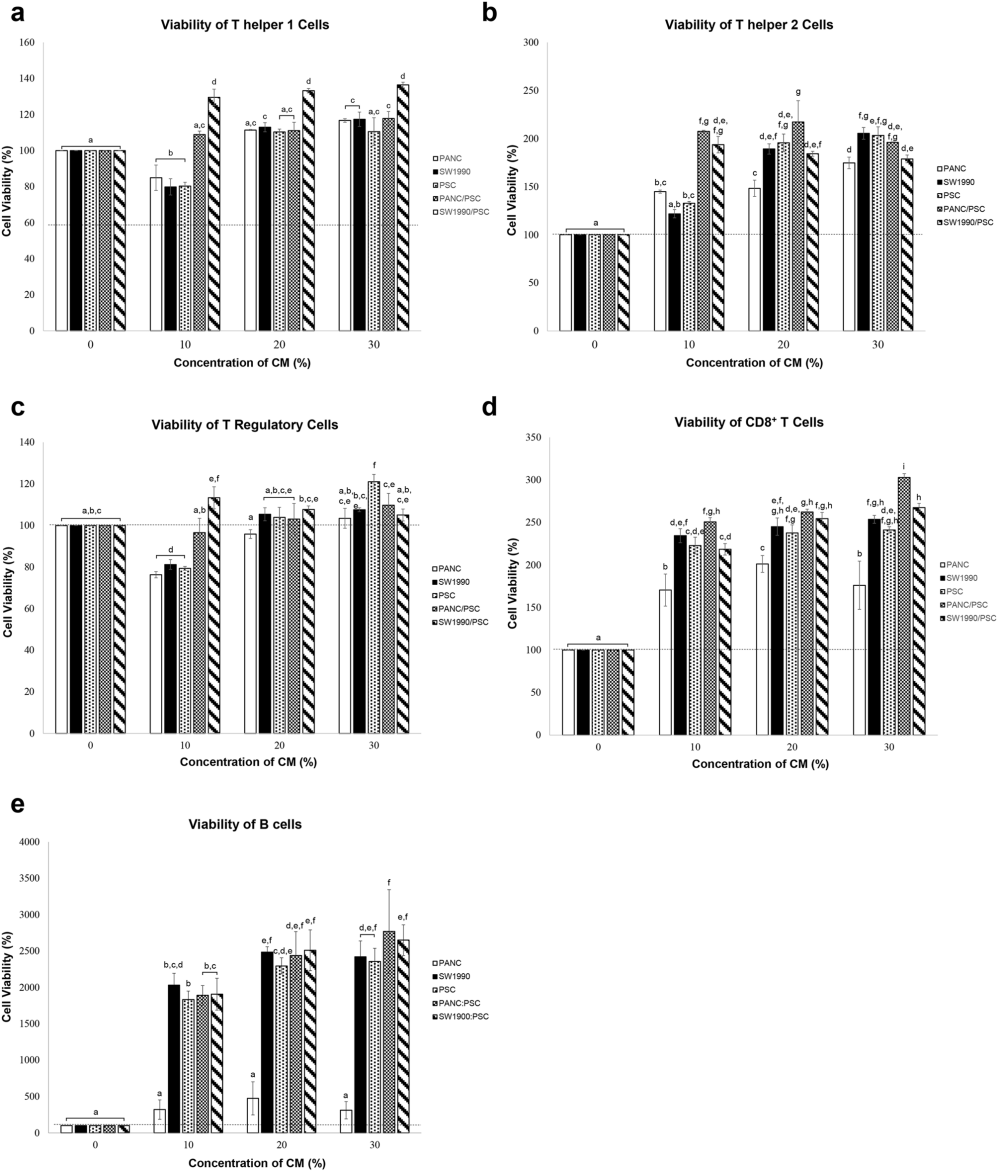
The viability of lymphocyte subtypes treated with medium conditioned by PCCs and PSCs for 48 h. (**a**) Th1, (**b**) Th2, (**c**) Treg, (**d**) CD8^+^ T cells, and (**e**) B cells. Statistical significance is indicated by the letters above each column, in which the columns that do not share a common letter have a significance of p ≤ 0.05.

**Figure 4:**
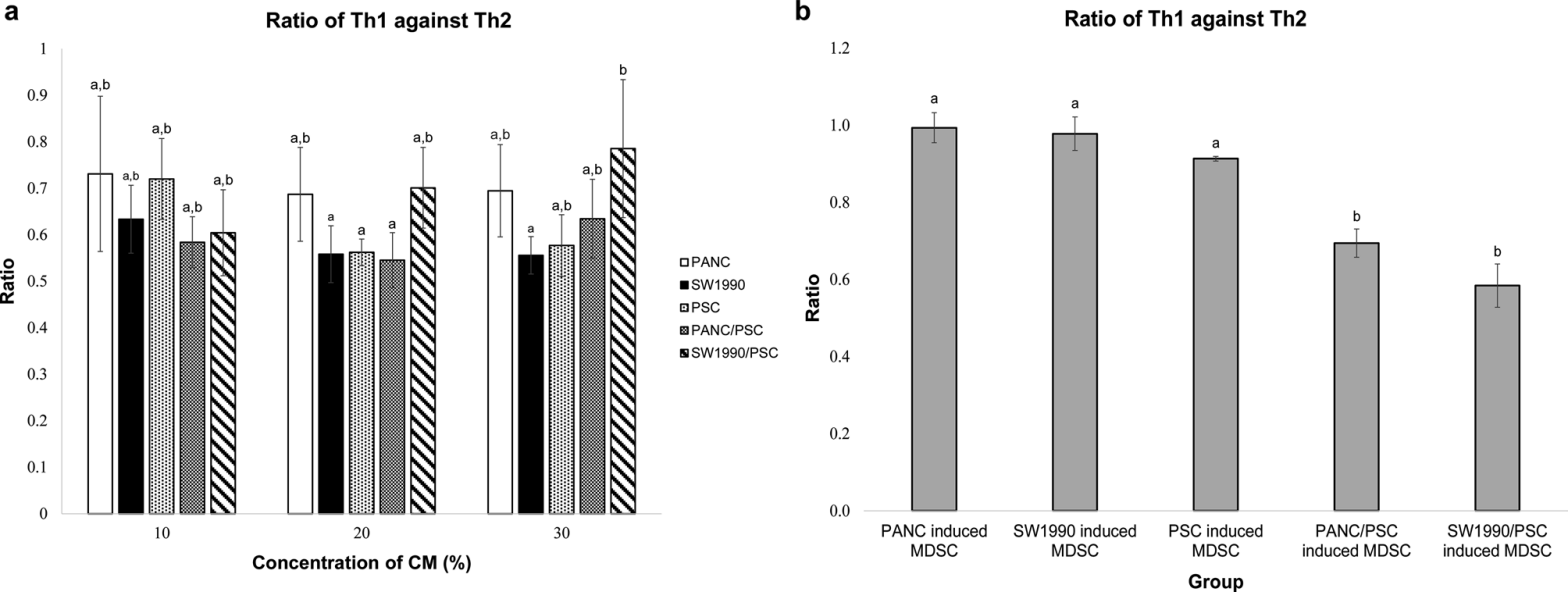
The ratio of Th1 against Th2. The ratio has been calculated based on the viability of Th1 and Th2 after treated with (**a**) CM and (**b**) CM-induced MDSCs for 48 h. Statistical significance is indicated by the letters above each column, in which the columns that do not share a common letter have a significance of p ≤ 0.05.

As shown in Fig. 3a, Th1 treated with 10% monocultures CM had a significantly lower viability (at least 20%) than untreated. Whereas for the co-cultures, Th1 treated with PANC10.05/PSC CM was not significantly different from untreated, but SW1990/PSC CM treated group had a viability that was about 30% higher than untreated. As the concentration of CM increased, the viability of all monocultures increased significantly while both co-cultures remained to be the same as 10% CM. For Th2 treated with CM, both co-cultures treated groups had a viability that was at least 30% higher than the monocultures and 100% higher than untreated (Fig. 3b). At 20% CM, all groups except PANC10.05 monoculture had achieved a similar level of Th2 viability. As the concentration of CM increased to 30%, all groups including PANC10.05 had achieved a similar viability. In addition, the ratio of Th1 against Th2 (Th1:Th2) was determined to investigate the importance of T helper cells balance in the TME of PDAC. As shown in Fig. 4a, the Th1:Th2 ratios of all groups were smaller than 1, indicating a higher proportion of Th2 than Th1.

For Treg, the viabilities of all monoculture’s CM treated groups were significantly lower than untreated and co-culture treated groups at 10% CM (Fig. 3c). At 30% CM, all groups had a viability that was not significantly different from the untreated group, except for PSCs monoculture, which had a viability that was about 20% higher.

As for CD8^+^ T cells, the viability for all treated groups increased significantly at a CM concentration as low as 10% (Fig. 3d). As compared to untreated, the viabilities of all treated groups were at least 70% higher, with the highest viability in PANC10.05/PSC CM treated group, which was about 200% higher. Furthermore, the viability of PANC10.05 monoculture treated group remained to be the lowest in all concentrations. However, when co-cultured with PSCs, PANC10.05/PSC had a viability that was about 70% higher than its monoculture.

Lastly, Fig. 3e shows the viability of B cells, in which the viability of PANC10.05 monoculture treated group remained to be the lowest in all concentrations. However, in the presence of PSCs, PANC10.05/PSCs co-culture treated group had achieved the highest viability, which was about 900% higher than its monoculture and over 2000% higher than untreated at 30% CM. Whereas for SW1990, the viability of its monoculture was at least 500% higher than PANC10.05, and no significant difference was observed between its mono- and co-culture treated groups. In short, suppression was not observed in any of the lymphocyte subtypes, suggesting that the secreted proteins of PCC and/or PSC did not have a direct suppressive effect on the lymphocytes.

### Effects of CM-induced MDSC on isolated T lymphocytes

As CM do not have a direct suppressive effect towards lymphocyte subtypes, we further isolated MDSCs induced by PCCs and PSCs CM to investigate if the suppression that we have observed in section “Effects of PCCs and PSCs CM on lymphocytes populations” is contributed by CM indirectly, via inducing the differentiation of MDSCs.

**Figure 5:**
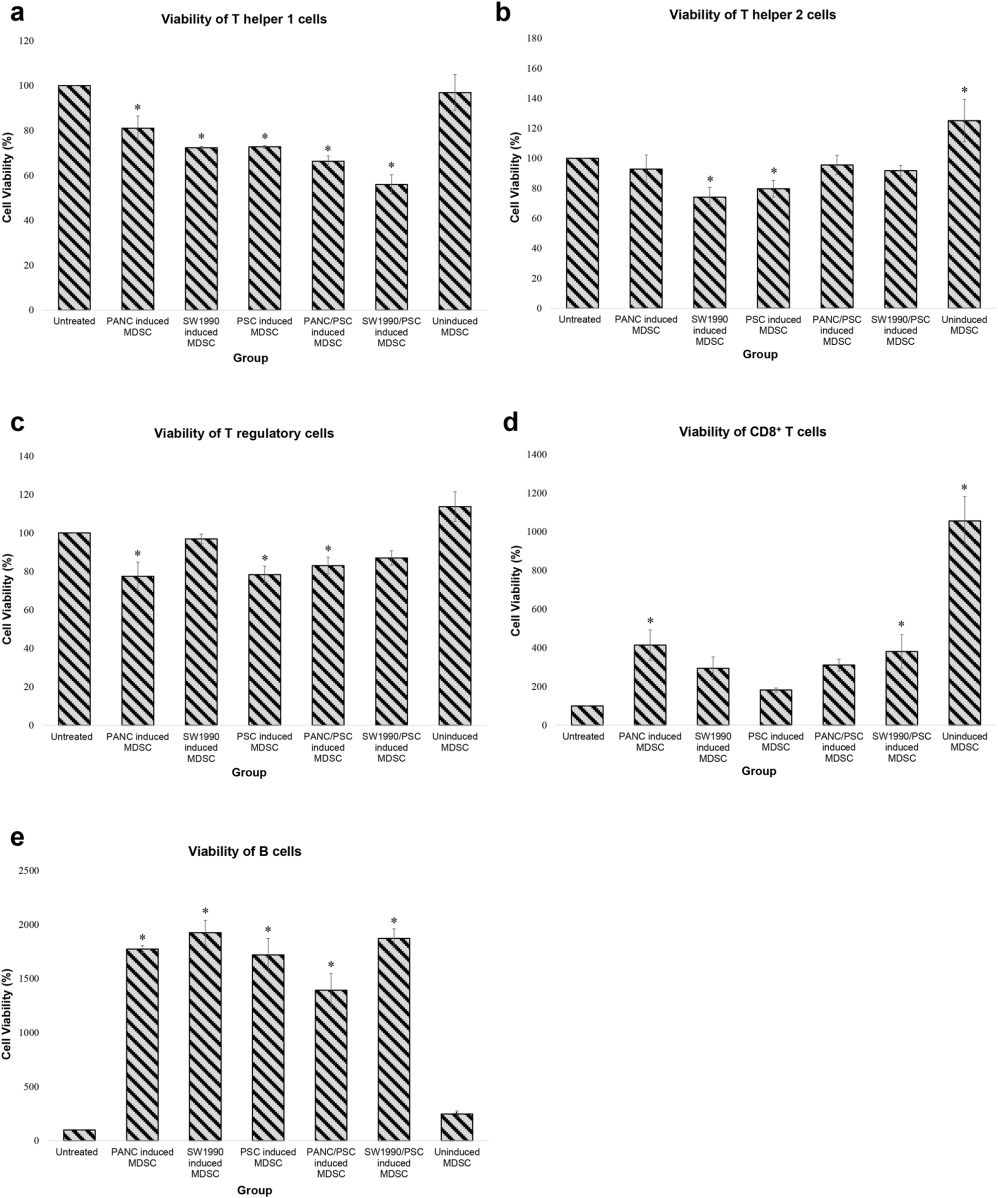
The viability of lymphocyte subtypes treated with MDSCs induced by PCCs and PSCs CM for 48 h. (**a**) Th1, (**b**) Th2, (**c**) Treg, (**d**) CD8^+^ T cells, and (**e**) B cells. Statistical significance is indicated by the asterisk above each column, in which the columns that have an asterisk represent that it has a significance of p ≤ 0.05 as compared to the untreated group.

According to Fig. 5a, Th1 treated with all groups of CM-induced MDSCs resulted in a viability that was at least 20% lower than untreated. As we compare the viabilities of monocultures, Th1 treated with MDSCs induced by PANC10.05 monoculture CM had a viability that was about 10% higher than SW1990 and PSCs monoculture treated groups. Whereas for the co-cultures, Th1 treated with PANC10.05/PSC CM-induced MDSCs had a viability that was about 20% higher than Th1 treated with SW1990/PSC CM-induced MDSCs. The uninduced MDSCs did not show significant suppression towards Th1, and the viability was not significantly different from untreated. For Th2, groups treated with SW1990 and PSC CM-induced MDSCs had a viability that was 20% lower than untreated (Fig. 5b). However, groups treated with PANC10.05 and both co-cultures CM-induced MDSCs did not show any significant difference in viability.

Similar to CM treated groups, the Th1:Th2 ratio in CM-induced MDSCs treated group was determined. According to Fig. 4b, all monoculture-induced MDSCs resulted in a ratio slightly lower than 1, indicating similar proportions of Th1 and Th2. Whereas for the co-cultures treated groups, the Th1:Th2 ratios were at least 40% lower than monocultures treated groups. For Tregs, PANC10.05, PSC, and PANC10.05/PSC-induced MDSCs treated groups had a significantly lower viability (about 20% lower) than untreated, whereas the remaining groups were not significantly different from untreated (Fig. 5c).

As compared to the untreated control, the viability of CD8^+^ T cells increased significantly (at least 300%) upon treatment with PANC10.05- and SW1990/PSC-induced MDSCs, with the highest peak in uninduced MDSCs treated group, which was about 900% higher (Fig. 5d). Whereas for B cells, all groups that were treated with CM-induced MDSCs had a significantly higher viability than the untreated group (over 1000% higher), while uninduced MDSCs group was 150% higher than the untreated group (Fig. 5e). Taken together, CM could induce MDSCs that were suppressive towards the subtypes of CD4^+^ T cells, but not the CD8^+^ T cells and B cells. However, unlike the uninduced MDSCs, the CM-induced MDSCs were able to suppress the further proliferation of CD8^+^ T cells and B cells.

## Discussion

In the past decades, studies have been conducted to investigate the immunosuppression in pancreatic cancer, hoping to develop an effective therapy that inhibits immunosuppression and improves patient’s outcome. In this study, we have determined the mechanism of PCCs and PSCs CM in exerting lymphocytes suppression in vitro.

**Figure 6:**
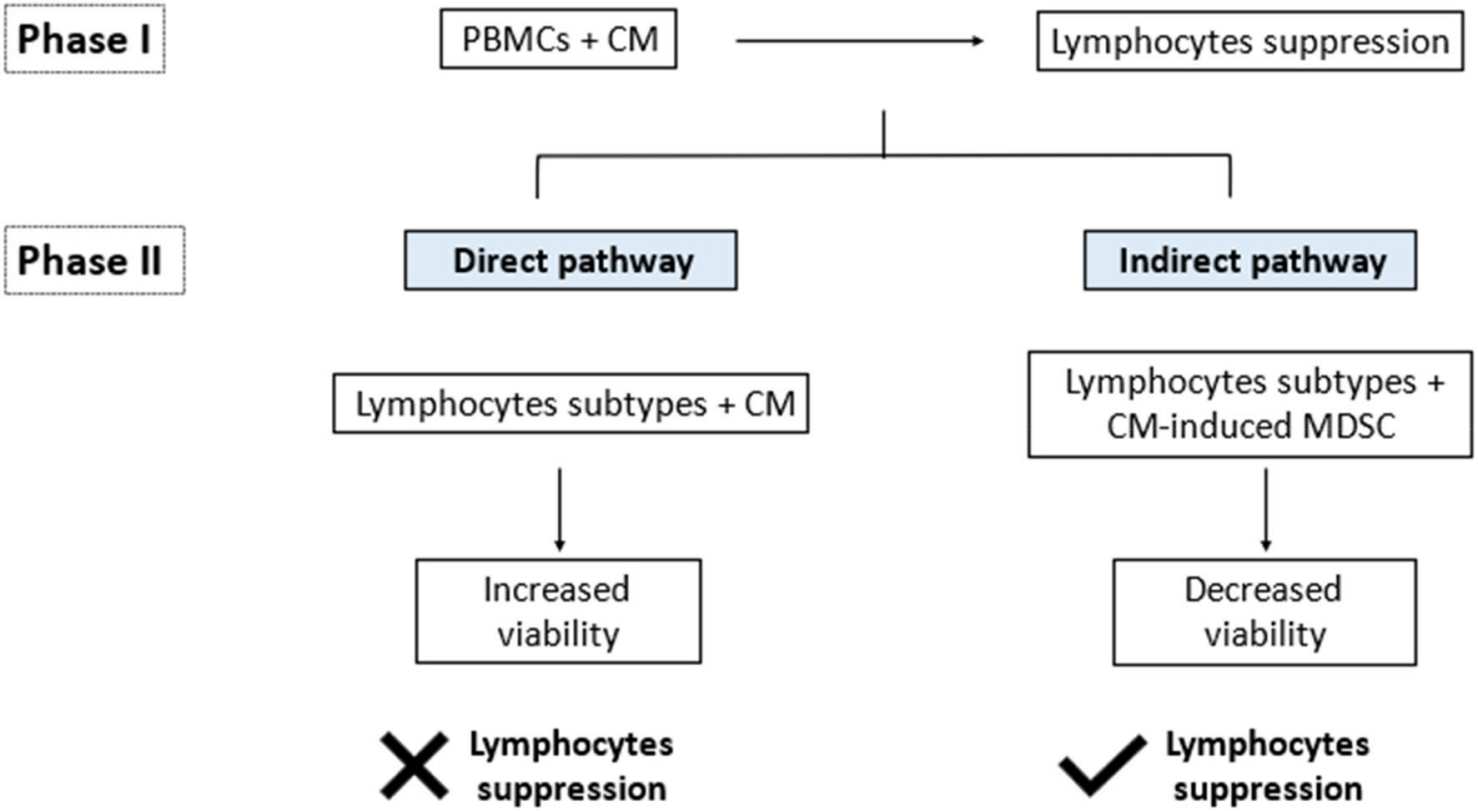
The summary of phase I and phase II. The study has been divided into two phases. Lymphocyte suppression was observed in the first phase. Whereas in the second phase, lymphocyte suppression was observed only in the indirect pathway.

In phase I, suppression of total lymphocyte population was observed in both PCCs and PSCs CM treated groups, as well as their co-cultures (Fig. 6). Without direct cell–cell contact, the secreted proteins by PCCs and PSCs can induce lymphocytes suppression. As shown by the significantly higher lymphocytes percentage, the lymphocytes suppressive effect exerted by the secreted proteins of the primary tumor-derived PCCs was proven to be weaker than the secreted proteins of the metastatic tumor-derived PCCs and PSCs. However, the suppressive effect can be enhanced in the presence of PSCs secreted proteins. Hence, we deduced that as PANC10.05 is established from a primary tumor; it required the interaction with PSC at the early stage of carcinogenesis to suppress antitumor immune response. As the tumor progresses, the grade II SW1990 cells that are established from a metastatic tumor could suppress the immune system independently. According to the proteomic analysis of CM, we hypothesize that the strong suppressive properties of SW1990 were contributed by the upregulated Transglutaminase 2 (TGM2). The expression of TGM2 was found to be upregulated in several types of cancer, which is associated with most of the highly aggressive forms of cancer^37^. TGM2 confers a strong protective role on cancer cells against apoptotic stresses and thereby promotes cancer cells survival^38^. Besides, TGM2 catalyzes the protein crosslinking and involves in multiple signaling pathways including the NF-κB signaling pathway, PI3K/Akt survival pathway, and TGF-β signaling pathway^37,39–41^. The immune suppressive role of TGF-β in PDAC has been widely reported, in which it inhibits the antitumor immunity of effector T cells and induces the immunosuppressive cell types, such as T regulatory cells (Tregs), T helper 2 cells (Th2) or tumor-associated macrophages (TAMs)^24,42–44^. Hence, we suggest that the upregulated TGM2 is one of the key players that confers the stronger suppressive properties of SW1990.

In phase I, we observed a significant suppression of total lymphocyte population induced by the secreted proteins in CM. As the secreted proteins of PCCs and/or PSCs are potent in inducing MDSCs differentiation, we hypothesize that MDSCs could be the key player that induces the lymphocytes suppression that we had observed in phase I^26,30^. Hence, we investigated the mechanism of CM in lymphocytes suppression via two different pathways in phase II (Fig. 6). In the direct pathway, different lymphocyte subtypes were isolated and treated with CM directly. Three concentrations of CM were used to simulate different stages of PDAC. The lower CM concentration (10%) simulates the early stage of PDAC, in which the low number of PCCs and PSCs limits the amount of secreted proteins available in the TME. Whereas the 20% and 30% CM simulate more advanced stages of PDAC, which have more PCCs and PSCs to enrich the TME with cytokines and other secreted proteins. Whereas in the indirect pathway, lymphocyte subtypes were isolated and treated with MDSCs induced by the CM of PCCs and/or PSCs.

The viability of T helper 1 cells (Th1) had increased significantly in all groups after being treated with CM, except for 10% monoculture CM treated groups. The precursor of Th1, naïve CD4^+^ T cells, polarize into different lineages and play a pivotal role in the activation and maintenance of the effector cells. Th1 is mediated by pro-inflammatory cytokines, and it promotes antitumor cellular immune response by activating CD8^+^ cytotoxic T cells, secreting IFN-γ that has a direct cytotoxic effect on PCC, and inducing humoral immune response through CD40 ligand signaling^45,46^. According to our results, we postulated that in the early stage of PDAC (at low CM concentration), monocultures secreted proteins can suppress the viability of Th1, thus reducing antitumor immune response. However, their co-culture secreted proteins are immunogenic and trigger the proliferation of Th1 even at low CM concentration, showing that the interplay between PCCs and PSCs is important as it promotes antitumor immune response. According to literatures, tumors regularly provoke adaptive immune response against tumor antigens are known to be immunogenic, such as the CD8^+^ T cell-mediated responses, although the majority were also self-antigens^47,48^. Immunogenic tumors have significant numbers of infiltrate immune cells and upregulated immune network, which can be either immune-suppressive or non-suppressive^23^. Hence, the secreted proteins that increase the lymphocyte viability were said to be immunogenic, as they can trigger the activation and proliferation of lymphocyte subtypes, regardless of its suppressive nature. Of note, we have also found out that there is a maximum efficiency for each CM to activate Th1 proliferation. Once the threshold is reached, increment of CM concentration will not trigger further expansion of Th1. In the presence of PSCs, SW1990 cells were found to be more immunogenic than PANC10.05 cells, which is expected as the metastatic, well-differentiated PCCs will express more tumor-specific antigens than the primary tumor-derived PCCs.

Th2 (T helper 2 cell) is known to be pro-tumorigenic as it releases cytokines that promote the expansion of other immunosuppressive cells, such as TAM^25,45^. Besides, as the antitumor immune response mainly depends on cell mediated immunity, Th2 that promotes humoral immunity will reduce the efficiency of antitumor immune response and promote chronic inflammation^49^. According to our results, we postulated that the interplay between PCCs and PSCs is important for immunogenicity, especially at the early stage of PDAC, disregards of their cell of origin. Our data also shows that the PANC10.05 cells are less immunogenic than the SW1990 cells and PSCs, disregards if the immune response is pro-tumorigenic (Th2) or anti-tumorigenic (Th1).

In PDAC, the differentiation of T helper is skewed from Th1 to Th2, thus limiting the antitumor cell-mediated immune response^24,50,51^. However, instead of the absolute number of either Th1 or Th2, the balance between Th1 and Th2 in the TME is more relevant to the clinical outcomes, as high Th1:Th2 ratio correlates with prolonged survival^49,52^. According to our data, the proportion of Th2 is higher than Th1 in all CM treated groups. Namely, the balance of immune response is tilted towards the pro-tumoral humoral immunity rather than antitumor cell mediated immunity. Other than affecting the balance of immune response, Th2 can also promote cancer cell growth, activate cancer-associated fibroblast that reduces the infiltration of immune cells, and induce the differentiation of TAM, which further enhances cancer progression^45,53^.

Tregs (T regulatory cells) act as an immune mediator in healthy individuals to prevent autoimmune diseases by suppressing immune response. In tumor immunology, Tregs were reported to be one of the immunosuppressive cells that suppress antitumor immune response^54,55^. According to our results, a similar trend with Th1 was also observed in Tregs, where the monocultures secreted proteins suppressed Tregs viability in the early stage of PDAC. This shows that without the cell–cell interactions between PCCs and PSCs, both pro-tumorigenic (Tregs) and anti-tumorigenic (Th1) immune responses would be suppressed in early PDAC. However, the Tregs viability was generally unaffected by CM treatment at higher concentrations, which suggests that the direct induction of Tregs by PCCs and PSC secreted proteins is unlikely to be the primary mechanism responsible for immunosuppression in advanced PDAC.

Cytotoxic (CD8^+^) T cells are the main effector T cells responsible for tumor-specific cell mediated immunity. This function is carried out by, i. producing IFN-γ that can induce differentiation of effector cytotoxic T cells, in which IFN-γ is also responsible for induction of antigen-specific cytotoxic T cells that leads to expansion of memory cells that are effective during cancer recurrence, and ii. producing cytotoxic granule components, such as granzymes and perforin^43,46,56–58^. According to our data, the trend is consistent with the observation in Th1, Th2, and Tregs that the primary tumor derived PCCs secreted proteins being the least immunogenic for both anti-tumorigenic and pro-tumorigenic immune responses, but the immunogenicity can be greatly enhanced in the presence of PSCs. Notably, the increment of viability percentage in all groups was much larger compared to the effect size observed in Th1 and Th2, which is an indication that the proteins secreted by PCCs and PSCs are more immunogenic towards CD8^+^ T cells than T helper cells.

The role of B cells in tumor immunology has remained unclear, as they have contradictory roles in tumor immunology. B cells promote antitumor immune response by being an antigen-presenting cell (APC) that enhances the expansion of antigen specific CD4^+^ and CD8^+^ T cells; on the other hand, they reduce the secretion of Th1 cytokines and impair cytotoxic (CD8^+^) T cells response^59,60^. According to the result, the effect size in the increment of viability was the greatest among all lymphocyte subtypes (at least 1800% higher than untreated). Hence, we hypothesized that 1. the secreted proteins of PCCs and PSCs have strong effects on effector lymphocytes proliferation especially B cells, 2. the induction of B cells division may be the primary mechanism responsible for PDAC immunosuppression, 3. the interaction with PSCs is necessary for the primary tumor-derived PCCs to trigger the pro-tumoral humoral immunity but not the metastatic tumor-derived PCCs, thus efforts targeting the interaction between PCCs and PSCs to reverse the immunosuppressive TME may be useful in primary tumor but futile in metastatic tumor. However, further study is required to validate this finding.

As lymphocytes suppression observed in flow cytometry analysis is not contributed by the secreted proteins of PCCs and PSCs directly, we deduced it may be caused by the MDSCs differentiated from PBMCs upon exposure to CM. To test this hypothesis, the MDSCs that were induced by PCCs and PSCs CM were isolated and co-cultured with various lymphocyte subtypes. According to the DEPs (Table 1), TGM2 and lipocalin 2 (LCN2) have been reported to associate with the accumulation of MDSCs^61–63^. Of note, the role of these proteins in MDSC differentiation has not been fully established, and further studies might be required for validation. According to the results, all groups of CM-induced MDSCs were suppressive against Th1, in which both co-cultures displayed lower Th1 viability than monocultures, suggesting that PCCs and PSCs work synergistically in Th1 suppression. Besides, in order to confirm that the proteins secreted by PCCs and/or PSCs are necessary to activate the suppressive MDSCs, we have isolated uninduced MDSCs from PBMCs (without CM treatment) and accessed its ability to exert lymphocytes suppression. The result shows that the uninduced MDSCs did not affect Th1 viability, as the viability of Th1 treated with uninduced MDSCs was not significantly different from untreated Th1. This data suggests that without CM induction, the uninduced MDSCs do not possess the ability to suppress Th1. Whereas for Th2, although SW1990 and PSCs CM-induced MDSCs resulted in a lower Th2 viability, while the uninduced MDSCs resulted in a higher viability. Notably, the effect size was only about 20%. Hence, we hypothesized MDSCs do not play a major role in the viability of Th2.

The Th1:Th2 ratio was also calculated for CM-induced MDSCs treated groups. Both co-cultures treated groups displayed a lower Th1:Th2 ratio than the monocultures treated groups. This shows that PCCs and PSCs work synergistically in differentiating MDSCs that reduce Th1:Th2 ratio, wherein promoting humoral immune response that is pro-tumoral. Besides, although not statistically significant, the SW1990 and PSC co-culture-induced MDSCs resulted in a slightly lower Th1:Th2 ratio than the PANC10.05 and PSC co-culture-induced MDSC, which might reflect a stronger pro-tumoral immune response in the metastatic PDAC.

According to the results, PANC10.05, PSCs, and their co-cultures CM-induced MDSCs are suppressive towards Tregs. As Tregs can suppress effective antitumor immune responses, thereby promote tumor development and progression^64^, we deduced that the suppressed Tregs by the PANC10.05 cells and PSCs leads to the better clinical outcome in early PDAC. Whereas in the advanced stage of PDAC, the SW1990 cells induce MDSCs that are not suppressive against Tregs will ultimately lead to cancer progression as Tregs suppress other antitumor immune responses. However, it is noteworthy that the observed effect size was small (20%).

For CD8^+^ T cells, instead of suppressed cells viability, the CD8^+^ T cells viability of all treated groups had increased by at least 80%. Noteworthy, the effect size of viability increment in CD8^+^ T cells treated with uninduced MDSCs was much larger than the other lymphocyte subtypes treated with uninduced MDSCs. Not only that it shows MDSCs without CM induction were not suppressive, but they are also having a strong effect in promoting the proliferation of CD8^+^ T cells. However, CM-induced MDSCs resulted in a smaller increment of CD8^+^ T cell viability compared with uninduced MDSCs. To our knowledge, this is the first report of the ability of MDSCs to induce the proliferation of CD8^+^ T cells, showing that MDSCs do not only play suppressive roles. The circulating MDSCs (immature and undifferentiated) from the PBMCs of healthy individuals could induce CD8^+^ T cells proliferation. However, when the MDSCs were differentiated and mature in vicinity to PCCs and PSCs, their ability to induce CD8^+^ T cells proliferation reduces. We deduce that if the incubation period was longer or higher concentration of CM were used, we would see suppressive nature of the MDSCs that was reported in other studies. Furthermore, it is unclear about the functionality of proliferated CD8^+^ T cells, such as their ability to release cytotoxic molecules.

As for the viability of B cells after treated with CM-induced MDSCs, the effect size of increment in the viability was even larger than the increment observed in CD8^+^ T cells. Some studies have proven that MDSC could increase the proliferation of B cell and produce antibodies that inactivate T cell responses^65–67^. As humoral immune response is not an effective anti-cancer immune response, we hypothesized that the huge increment in B cells viability could contribute to cancer progression by tilting the immune response towards humoral rather than cell-mediated immunity. This observation is in line with the results observed in Th1, Th2 and Th1:Th2 ratio (Figs. 4b, 5a,b).

**Figure 7:**
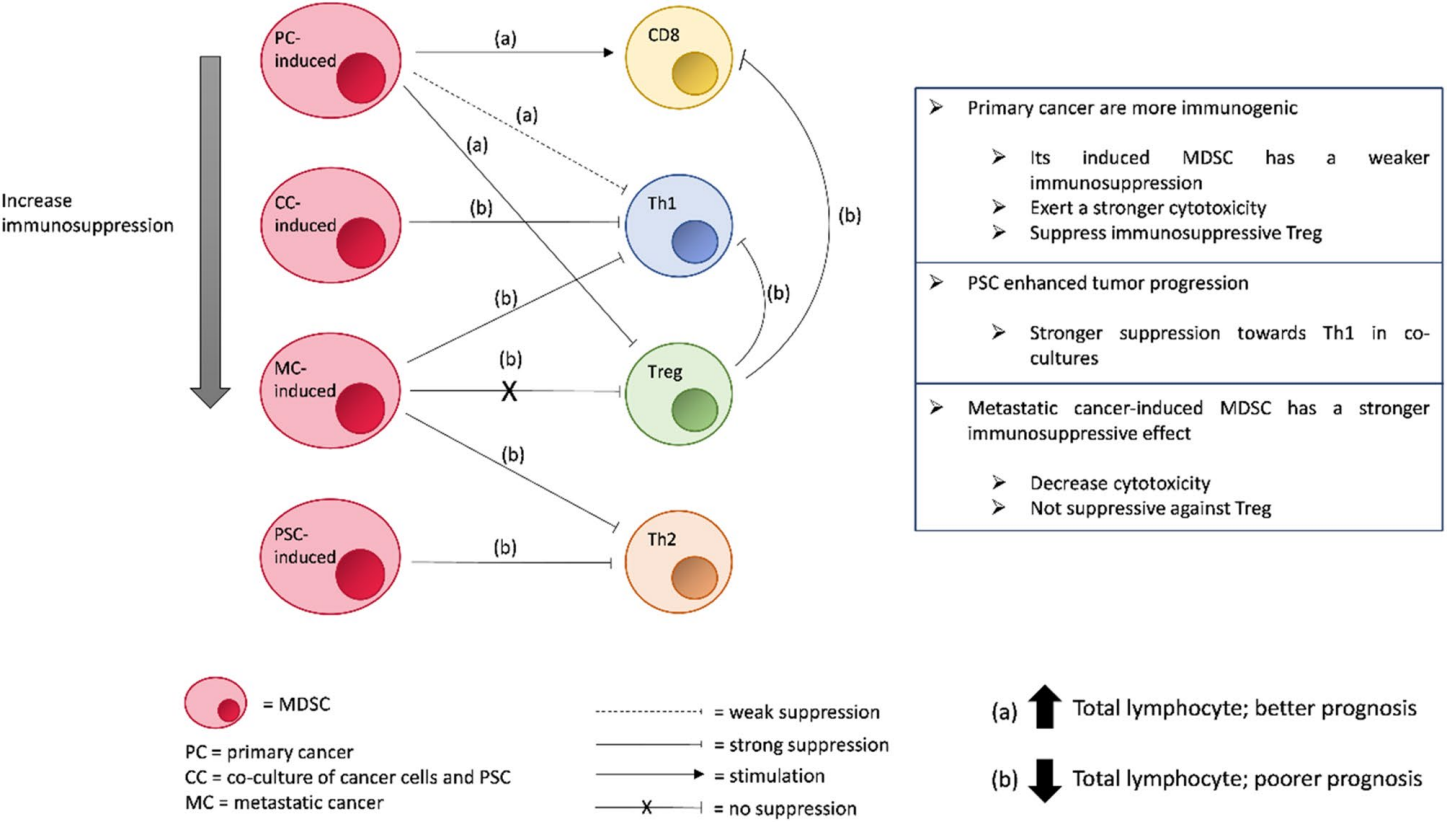
The effects of CM-induced MDSCs on each T cell subtype.

In phase I, it was found that when PBMCs were treated with CM, the total lymphocytes of all groups were greatly suppressed, and we observed a different intensity of suppression between the PCC lines that were derived from different stages of tumor. This led to our further investigation on the direct and indirect effects of CM towards each lymphocyte subtype, and what contributes to the milder suppressive effect exerted by the primary tumor-derived PCCs. To provide a better visualization for the hypothesis that we have made based on the results from phase I and phase II, the complex interplays between lymphocyte subtypes and CM-induced MDSCs are as shown in Fig. 7. The primary tumor-derived PCCs had a weaker total lymphocytes suppressive effect than the metastatic tumor-derived PCCs due to (1) weaker Th1 suppression, (2) higher CD8^+^ T cells expansion, and (3) stronger Tregs suppression^44,55,68^. Eventually, the combinatory effects resulted in a weaker lymphocytes suppressive effect, thus leading to the better anti-cancer response and better prognosis in the early stage of PDAC. On the contrary, the metastatic tumor-derived PCC-induced MDSCs exhibit strong total lymphocytes suppression due to, (i) stronger Th1 suppression, (ii) stronger Th2 suppression, and (iii) no Treg suppression. The combinatory effects will then lead to suppressed antitumor immune responses with poorer prognosis in the advanced stage of PDAC. In accordance with a published work, Trovato et al. had reported that MDSCs isolated from patients with different stages of pancreatic cancer possessed a different degree of immunosuppression, which is not correlated to the MDSC subtype but the genomic and transcriptomic profiles^69^. This report is in line with our results that the MDSCs induced by the CM of SW1990 cells had stronger pro-tumoral characteristics. Lastly, despite the PSC-induced MDSCs that are strongly suppressive against Th2 that promote pro-tumoral immune response, a strong suppression towards Th1 was observed when PSCs are co-cultured with PCCs. This is an indication that the co-culture of PCCs and PSCs is important as it enhances the suppression towards antitumor immune response, disregards if it is in early or advanced stage of PDAC.

## Conclusion

A limitation of this study is that the lymphocyte subtypes were isolated and treated with CM and CM-induced MDSCs separately. Hence, the interactions between immune cells will not be observed, such as the suppression exerted by Tregs on antitumor immune cells, and the overview of cell–cell interaction in Fig. 7 is deduced based on our results and literature review. Nonetheless, a direct co-culture of lymphocyte subtypes with CM or CM-induced MDSCs will clarify the relationship between immune cells, facilitating the discovery of the underlying mechanisms. Furthermore, the bioactive secreted proteins in the CM should be identified as they may serve as potential targets for pancreatic cancer immunotherapy. In conclusion, CM did not have direct suppressive effects against any of the lymphocyte subtype. However, the MDSCs induced by CM of different cancer stages exhibited a different degree of lymphocytes suppression. Besides, the co-culture of PCCs and PSCs showed significant difference in their suppressive effects as compared to their monocultures. Hence, the co-culture should be included in future studies to better mimic the TME of PDAC.

## Data Availability

The datasets used and/or analyzed during the current study are not publicly available due to individual privacy concern but are available from the corresponding author on reasonable request.

## Abbreviations

PDAC: Pancreatic ductal adenocarcinoma
PCC: Pancreatic cancer cell
PSC: Pancreatic stellate cell
MDSC: Myeloid-derived suppressor cell
ECM: Extracellular matrix
TME: Tumor microenvironment
PBMC: Peripheral blood mononuclear cell
IL: Interleukin
GM-CSF: Granulocyte–macrophage colony-stimulating factor
VEGF: Vascular endothelial growth factor
7AAD: 7-Aminoactinomycin D
CM: Conditioned medium
Th1: T helper 1 cell
Th2: T helper 2 cell
Treg: T regulatory cell
TGF-β: Tumor growth factor-β
IFN-γ: Interferon-γ
DEP: Differentially expressed protein
DTT: Dithiothreitol
IAM: Iodoacetamide
FDR: False discovery rate
FC: Fold change
GO: Gene ontology
TGM2: Transglutaminase 2
LCN2: Lipocalin 2
