# Author Correction: Conditioned media of pancreatic cancer cells and pancreatic stellate cells induce myeloid-derived suppressor cells differentiation and lymphocytes suppression

**DOI:** 10.1038/s41598-023-40226-1

**Published:** 2023-08-10

**Authors:** Yuen Ping Chong, Evelyn Priya Peter, Feon Jia Ming Lee, Chu Mun Chan, Shereen Chai, Lorni Poh Chou Ling, Eng Lai Tan, Sook Han Ng, Atsushi Masamune, Siti Aisyah Abd Ghafar, Norsharina Ismail, Ket Li Ho

**Affiliations:** 1 School of Postgraduate Studies, International Medical University, Kuala Lumpur, Malaysia; 2 School of Pharmacy, International Medical University, No. 126, Jalan Jalil Perkasa 19, Bukit Jalil, 57000 Kuala Lumpur, Malaysia; 3 Division of Gastroenterology, Tohoku University Graduate School of Medicine, Sendai, Japan; 4 Department of Basic Science and Oral Biology, Faculty of Dentistry, Universiti Sains Islam Malaysia, Seremban, Malaysia; 5 Natural Medicines and Products Research Laboratory, Institute of Bioscience, Universiti Putra Malaysia, Serdang, Selangor Malaysia

Correction to: *Scientific Reports* https://doi.org/10.1038/s41598-022-16671-9, published online 19 July 2022

The Funding section in the original version of this Article was incomplete. It now reads:

“The study is funded by the Fundamental Research Grant Scheme (FRGS), Malaysian Ministry of Higher Education. Grant number FRGS/1/2018/SKK08/IMU/03/2.”

The original Article has been corrected.

